# Independent Component Analysis and Decision Trees for ECG Holter Recording De-Noising

**DOI:** 10.1371/journal.pone.0098450

**Published:** 2014-06-06

**Authors:** Jakub Kuzilek, Vaclav Kremen, Filip Soucek, Lenka Lhotska

**Affiliations:** 1 Department of Cybernetics, FEE, CTU in Prague, Prague, Czech Republic; 2 Czech Institute of Informatics, Robotics, and Cybernetics, CTU in Prague, Prague, Czech Republic; 3 Department of Cardiovascular Diseases, ICRC, St. Anne's Hospital in Brno, Brno, Czech Republic; College of Mechatronics and Automation, National University of Defense Technology, China

## Abstract

We have developed a method focusing on ECG signal de-noising using Independent component analysis (ICA). This approach combines JADE source separation and binary decision tree for identification and subsequent ECG noise removal. In order to to test the efficiency of this method comparison to standard filtering a wavelet- based de-noising method was used. Freely data available at Physionet medical data storage were evaluated. Evaluation criteria was root mean square error (RMSE) between original ECG and filtered data contaminated with artificial noise. Proposed algorithm achieved comparable result in terms of standard noises (power line interference, base line wander, EMG), but noticeably significantly better results were achieved when uncommon noise (electrode cable movement artefact) were compared.

## Introduction

In 1998 *Wisbec et al.* published a manuscript describing deployment of ICA on ECG obtained from 8 precordial electrodes [1]. Researchers deployed Fast ICA algorithm on ECG measured on 8 electrodes placed on chest. The separation of breathing artefacts into several independent components containing artefacts and minor portion of ECG activity was reported. In the same year *Barros et al.* [2] presented their contribution on ECG source separation using ICA neural network implementation. Simulation experiments were focused on measuring quality of separation against number of iterations required for the de-mixing matrix estimation.

Following these two pilot works in discussed field other researchers provided their solutions [3]–[15]. From those dealing with physiological signals we are listing some that of interest:

- *He et al.* [3] (2006) proposed an automatic method for EMG reduction based on JADE algorithm. The noise removal technique for selection of noisy components is based on thresholding of kurtosis and variance of components. Reported results showed that kurtosis of ECG activity is higher than kurtosis of EMG (in orders of magnitude) enabling EMG component identification and artefact reduction in resulting ECG.
- *Chawla et al.* [4] (2008) deployed JADE algorithm on three channel ECG. No comparable results were reported and the method is vaguely described, so the reproducibility of research is limited. This work employed kurtosis and variance for detection of noisy component in the same way as He et al. [3].
- *Milanesi et al.* [6] (2008) deployed FastICA and its modification for motion artefact removal from holter recordings. They studied ICA for convolutive mixtures and constrained ICA. The study proposes two measures of noise elimination – error estimate and correlation coefficients. It also employed statistical analysis of results obtained on data from 9 patients, which are over 5 minutes long.
- *Acharyya et al.* [11] (2010) deployed FastICA algorithm on MIT-BIH 3 channel ECG database in order to remove artefacts from electrocardiogram. They developed an algorithm for detection of component containing ECG based on Pearson correlation coefficient. This approach does not deal with signal reconstruction and noise reduction. The ECG morphology changes were not discussed.

There exist several works from area of functional magnetic resonance imaging (fMRI) strongly related with our work. *Thomas et. al* [16] proposed a solution for noise reduction of noise within BOLD-based fMRI using Principal and Independent Component Analysis (PCA and ICA). Their approach identified noise components using Fourier decomposition and removed found components from the data. This increased BOLD contrast sensitivity, which reflects the ability to detect BOLD signal within noise. ICA has been reported as good method for isolation of structured and random noise [16], while PCA was superior in isolation of random noise. Another work within fMRI field of research was reported by *Kiviniemi et. al.* [17]. The researchers used ICA for separation of spontaneous physiological sources in 15 anaesthetized children. The ICA was able to separate several signal clusters in the primary sensory areas in all subjects corresponding to vasomotor waves in fMRI data. The main purpose of this study was to source localization within the fMRI of brain. Last study related to our work was reported by *Liu et al.* [18]the researchers used Canonical Correlation Analysis (CCA) with Singular Value Decomposition (SVD) to reduce noise contained in fMRI. The method selects structured and unstructured CCA noise components and removes them from the data during the reconstruction process. The SNR of data was significantly improved by applied method [18].

In this paper we are presenting general approach suggesting that BSS algorithm can be easily replaced by current one. In our case we are working with JADE algorithm, which uses kurtosis for estimation of independent sources. Our approach combines BSS algorithm with detection step, which is similar to case in [16], [18]. Instead of searching for two types of noise (structured and unstructured) our method searches for the ECG containing components, marks them and then removes all other components. We also present results of extensive testing of developed method against two other approaches using large database containing 382 different ECGs from various databases available at [19]. Our method was primary developed for dealing with the electrode cable movement artefact, however we proved that it can be used for different types of noises.

The work described here is available online at http://bio.felk.cvut.cz/~kuziljak/.

### Data

For the evaluation experiments we used data freely available on MIT medical data storage Physionet [19]: Normal Sinus Rhythm database [19], European ST-T database [20], Long Term ST database [21], QT database [22], MIT Long Term database [19] and MIT-BIH ST Change database [23]. This gives us database containing 382 ECG recordings from different sources. All recordings were resampled to sampling frequency 500 Hz in order to make the evaluation easy to interpret.

## Methods

### Noise simulation

Various kinds of noise presented in ECG can be expected [24]. The most frequent noise types are as follows:

- Power line interference – consists of 50 Hz (60 Hz in U.S.) pickup and its harmonics, typical amplitude is up to 50 percent of peak-to-peak ECG amplitude.
- Muscle contraction – generates artefactual millivolt-level potentials, its standard deviation is around 10 percent of peak-to-peak ECG amplitude.
- Baseline drift and ECG amplitude modulation with respiration – is represented as slow sinusoidal component at respiration frequency, its amplitude variation is 15 percent of peak-to-peak ECG amplitude and typical frequencies at 0.15 Hz to 3 Hz.

In addition to these three types of noise we observed noise typically generated by electrode cable movement during ECG holter recording of ECG. This noise has large amplitudes up to 200 percent of peak-to-peak ECG amplitudes and typical power spectra containing peaks at 1.5, 3.16, 6.3 and 8 Hz.

In order to test performance of the algorithm we artificially added noises to ECG recordings. The noises were generated as follows:

- Electromyographic noise – is simulated as random Gaussian signal with deviation around 10 percent of peak-to-peak ECG amplitude.
- Power line interference −50 Hz sinusoid with amplitude 0.333 mV.
- Baseline wander – slow sinusoid (0.333 Hz) with the amplitude around 1 mV.
- Electrode cable movement – generated as sum of sinusoids with different amplitudes and frequencies ranging from 0.1 to 1 mV and 1.5 to 8 Hz respectively.

**Figure 1:**
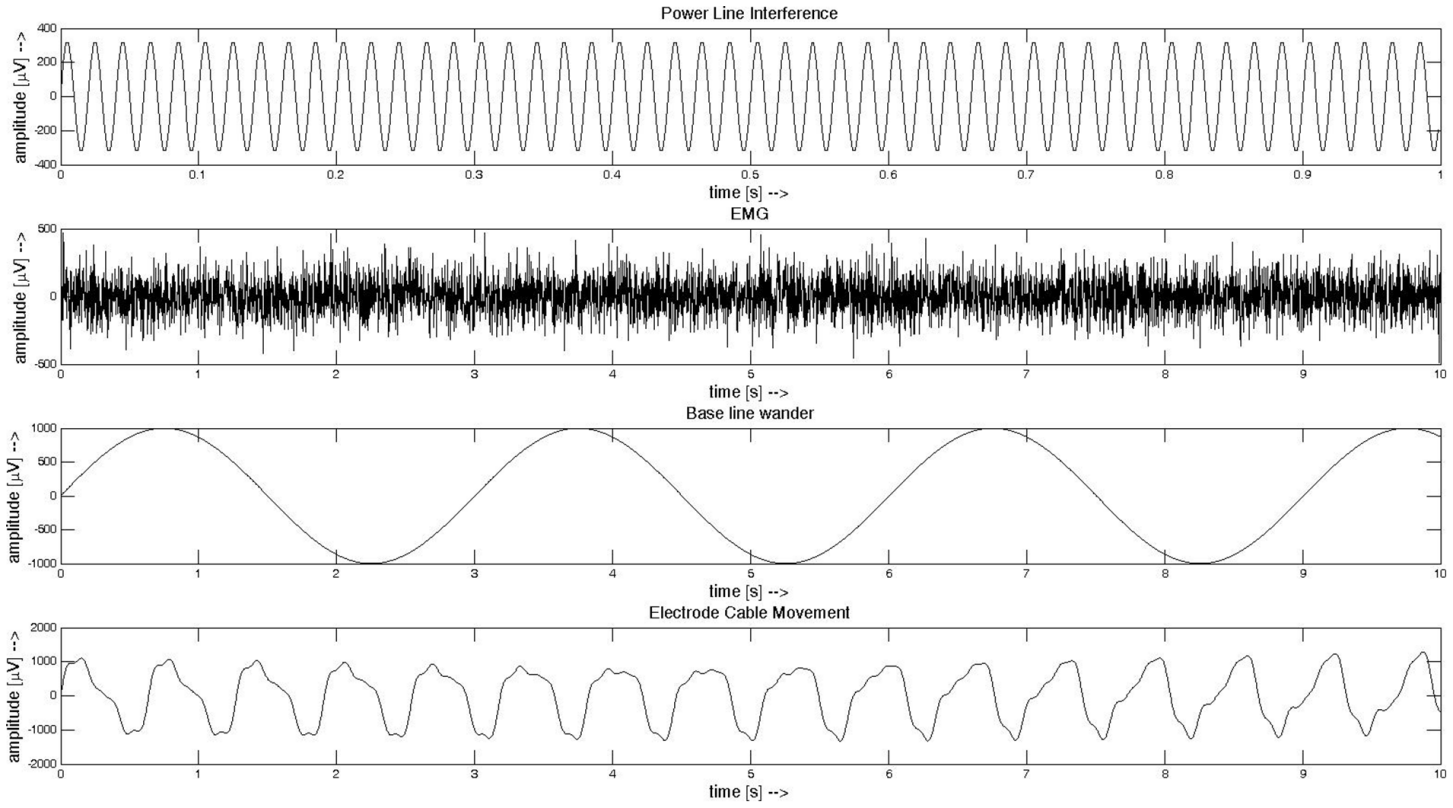
Examples of artefacts artificially added to ECG signals. From top to the bottom: 50 Hz power line interference, EMG, base line wander and electrode cable movement.

Each type of simulated noise is added to ECG at four different levels: 25, 50, 75, 100 percent of the maximum noise amplitude. The noise is added to each lead of ECG with different amplitude estimated from ECG amplitude. The examples of generated noises are shown in Fig. 1.

### Blind source separation

For “detection” of independent sources in our case – ECG and noise presented in ECG recordings, we used Independent Component Analysis (ICA), which one solution of the Blind Source Separation problem (BSS), which is the extraction of a set of signals based merely on their mixtures [25]. In particular let us mention ECG, which is a mixture of signals from nodes presented in the heart, or EEG, which is a mixture of neurological activity of centres in brain. Basic ICA model assumes linear combination of source signals (called components):
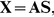
(1)where **X** is a mixture of source signals, **A** is the mixing matrix and **S** are the source signals. **X** and **S** get the size n x m, where n is number of sources and m is length of record in samples. Mixture matrix **A** is then of size n x n (in general **A** does not need to be square). Components can be obtained using the following expression:
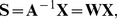
(2)where matrix **W** is inverse to matrix **A**. From Equation 2 it is obvious that estimation of a components is reduced to search of matrix **W**.

The BSS/ICA methods try to estimate components that would be as independent as possible and their linear combination is original data. Estimation of components is done by iterative algorithm, which maximizes function of independence, either by a non-iterative algorithm, which is based on joint diagonalization of correlation matrices.

In our method we used Joint Approximate Diagonalization of Eigen matrices algorithm (*JADE*) [25]. The algorithm is based on diagonalization of fourth-order cumulant tensor (for details see Appendix S1).

The problem, which emerges with holter ECG recordings, is low data dimensionality. Typically we are dealing with recordings with two or three leads. This may cause improper component estimation. In order to reduce the problem we applied technique described in [26], [27]. Briefly the main idea is to add time delayed data in to the dataset, forming new extended data set. To be more specific if the data contains less then 3 leads our method applies this approach for the data set extension.

### Features for component selection

For each estimated component selected features are computed, for details see Table 1. Those, which are chosen during training process of decision tree, are marked bold (see following Section).

**Table 1:** Features used for training decision tree using CART algorithm.

Feature	Definition	Additional information
Mean of component	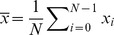	Value of component mean should be zero. We measured mean for both classes and its mean value was 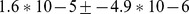 for non-noisy components and 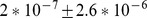 for noise containing components. Thus we decided to add this feature to the feature set.
Variance of component	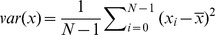	Value of component variance should be zero. We measured variance for both classes and its mean value was 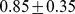 for non-noisy components and 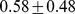 for noise containing components. Thus we decided to add this feature to the feature set.
**Kurtosis of component**	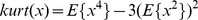	ECG has super-Gaussian distribution [3], [36], [37], thus it can be identified using kurtosis.
**Standard deviation of peak-to-peak distances**	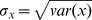	ECG has periodical rhythm, thus standard deviation of peaks detected by Pan-Tomkins detection algorithm [38] will be small.

Features left in decision tree after pruning are marked bold.

### Classification and regression tree

Classification And Regression Trees (CART) algorithm [28] is a classification algorithm for building a decision tree based on Gini's impurity index as splitting criterion (for details see Appendix S2).

CART tree needs to be trained before it can be used within our method. Tree training process automatically selects the most significant features and adjust the decision thresholds for them in order to increase the prediction accuracy (for details see Appendix S2).

**Figure 2:**
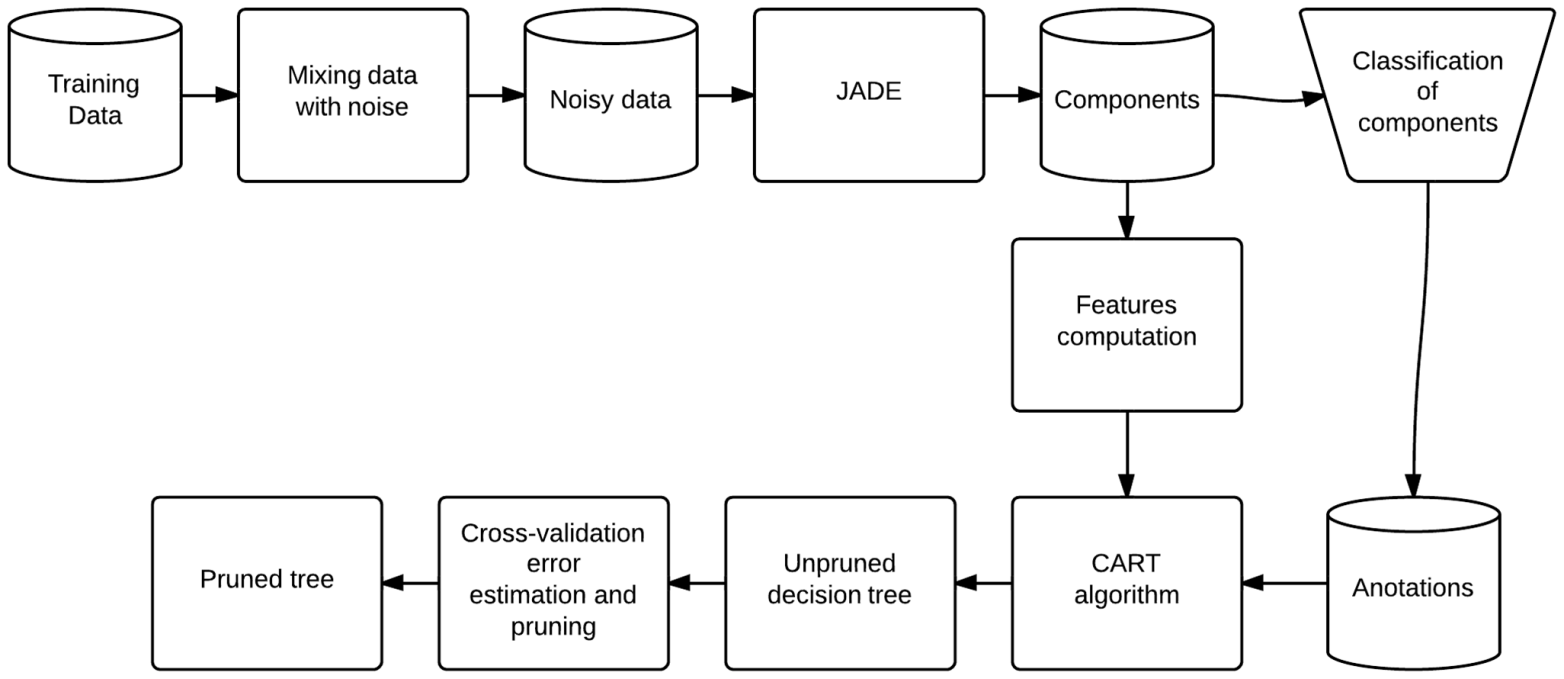
Decision tree training and pruning. First noise is added to the ECG data then components are estimated using JADE algorithm and components were manually labelled into noisy and noise-free groups. Features are computed and using them and annotations binary decision tree is trained using CART algorithm. Then the tree is passed to cross-validation and pruning and final pruned tree is created.

To train the tree we used data obtained from MIT-BIH Arrhytmia database [29], which contains 48 half-hour two channel ambulatory ECG recordings. All recordings from database were re-sampled to 500 Hz and 100% noise of each type is mixed to them. Then the components from each recording were estimated and visually classified as noisy/noise-free. Using features computed on classified components the decision tree was trained and pruned. The whole process of decision tree generation is shown in Fig. 2. The parameters of tree in Fig. 2 were determined by the CART learning algorithm. They have been selected as result of training process and the decision tree needs to be trained in case of new type of noise.

### Proposed algorithm

**Figure 3:**
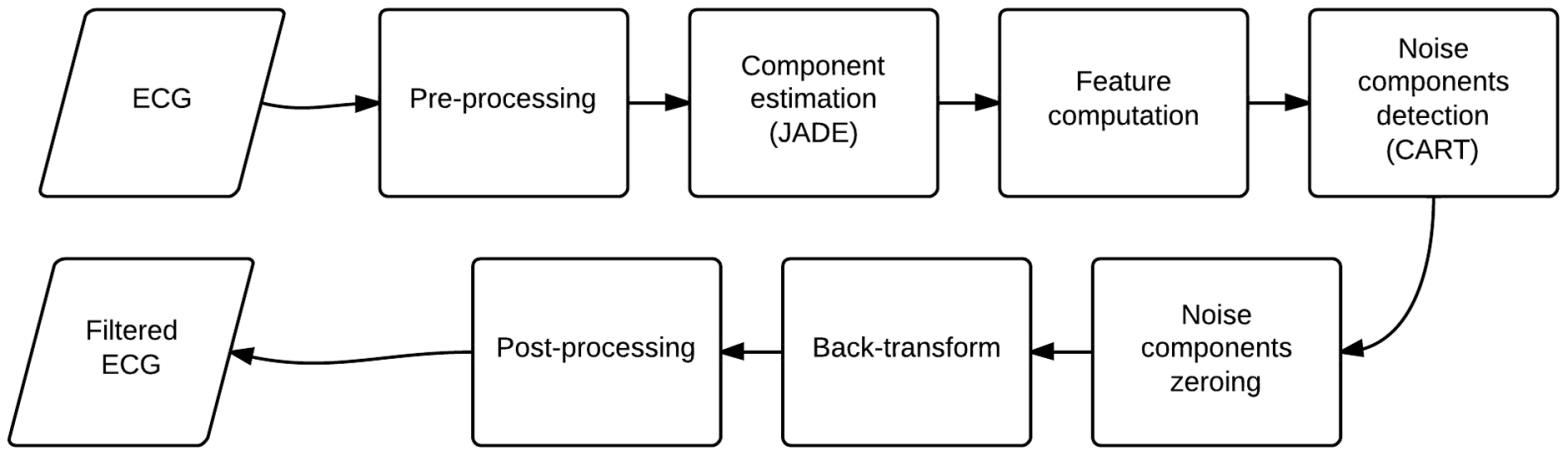
ICA based noise removal algorithm work-flow. First data is preprocessed, then the components are estimated. After that components containing noise are identified and removed. Finally ECG is reconstructed and filtered using post-processing filter.

Proposed algorithm combines JADE based source separation with CART decision tree in order to identify and remove noise from ECG. The algorithm work-flow is shown in Figure 3.

**Figure 4:**
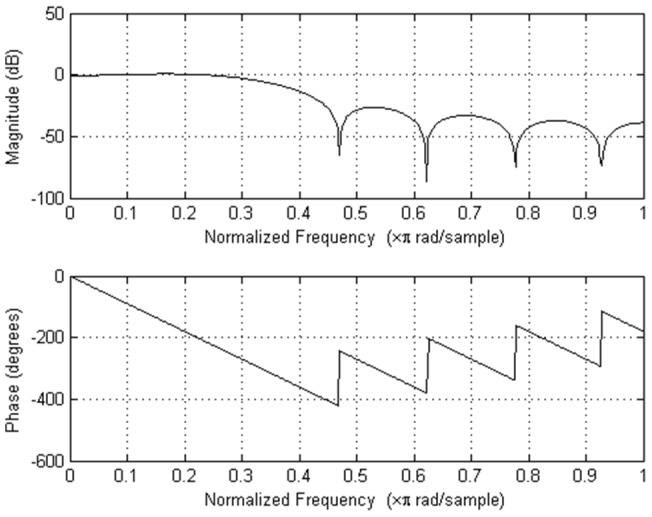
Frequency response of post-processing low pass FIR filter with first zero at 117 Hz, delay 5 samples and gain 0.93.

Firstly mean is subtracted from ECG recording. Then the independent components using JADE algorithm are estimated. For each component set of features were computed and features are passed to trained decision tree, which decides whether component is noise or ECG containing. Finally components marked as noise are removed, all signal are projected back to signal domain, filtered to remove high frequency noise (observed on 4 cases of training database) using low pass filter with first zero at 117 Hz, delay 5 samples and gain 0.93. Filter frequency response is shown in Fig. 4.

### Other de-noising techniques

Here we describe methods, which we used in order to evaluate the developed algorithm. According to our best knowledge, we chose proved and widely used methods.

#### Standard filtering algorithm

We merged basic noise removal techniques used in ECG signal processing in to one set. Each algorithm is applied on ECG signal in following order:

1. Adaptive noise canceller [30] for power line interference reduction.
2. Notch filter for EMG suppression [31].
3. Median filter for base line wander reduction [32].

#### Wavelet decomposition based algorithm

Wavelet de-noising is based on method described in [33]. The algorithm contains three steps:

• Wavelet decomposition using Daubechies wavelet DB6.
• Threshold detail coefficients using hard thresholding method [34].
• Wavelet reconstruction based on original approximation coefficients and modified detail coefficients [35].

### Evaluation

For evaluation we used Root-Mean-Square Error (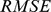), which is good statistical index for case, when the original clear signal is known. 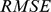 is defined as:
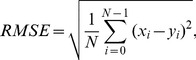
(3)where 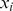 is the 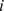-th sample of original signal, 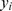 is the 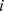-th sample of filtered signal and 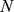 is the number of samples in both signals. 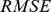 equal to zero means that original and filtered signal are identical.

**Figure 5:**
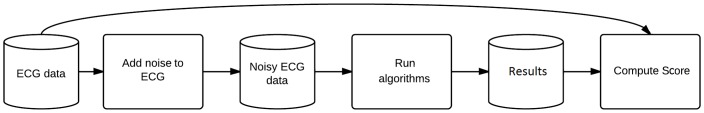
Evaluation process.

Evaluation process is depicted in Figure 5. First the data were mixed with the simulated noise in order to create noisy recordings with predefined level of noise. Then our method and other de-noising methods were applied on the data and the results were obtained. Finally resulting signals were compared to the original ECG data using 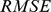 measure and the result is stored for the evaluation.

## Results

**Figure 6:**
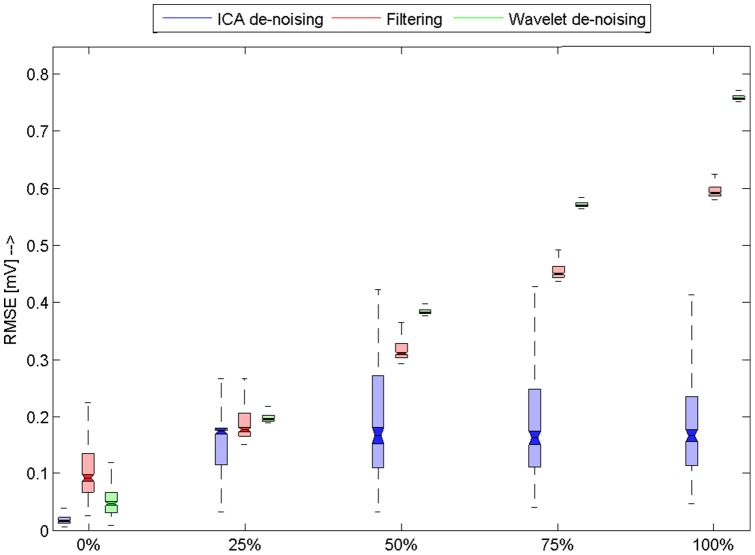
Results for the electrode cable movement artefact. Horizontal axis shows level of added noise. Boxplots shows RMSE for used database of signals. Filtered methods that were used are differentiated by colors. We can observe that ICA based method outperforms both referential methods and its results are significantly better, as it can be seen in figure.

**Figure 7:**
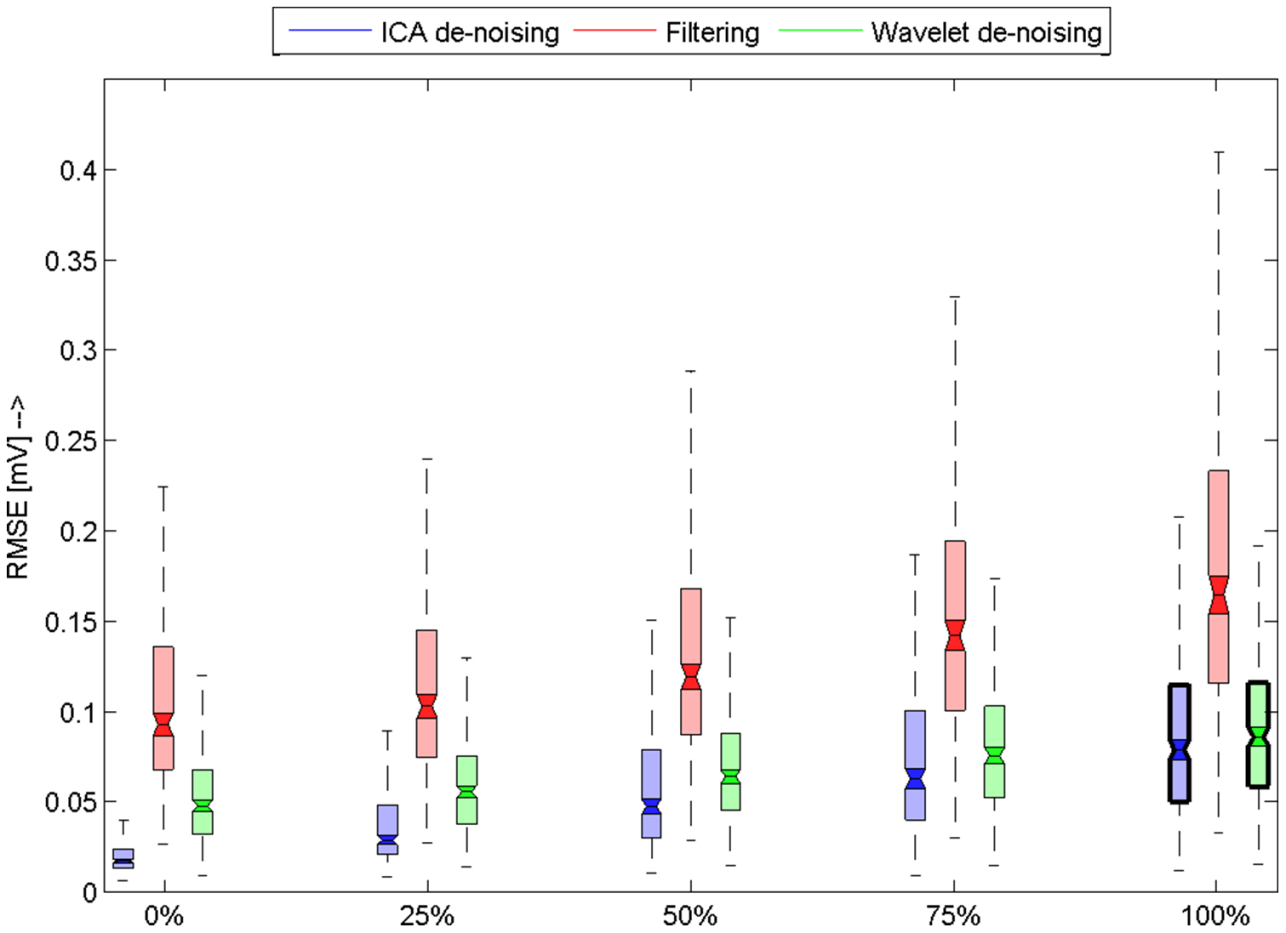
Results for EMG artefact. Horizontal axis shows level of added noise. Boxplots shows RMSE for used database of signals. Filtered methods that were used are differentiated by colors. Those boxplots were notches are marked with bold border around borders means that they means are not significantly different.We can observe that our methods is slightly better or similar to wavelet filtering method. Normal filtering method is slightly worse because of spectral characteristics of the simulated noise, which covers large interval of frequencies.

**Figure 8:**
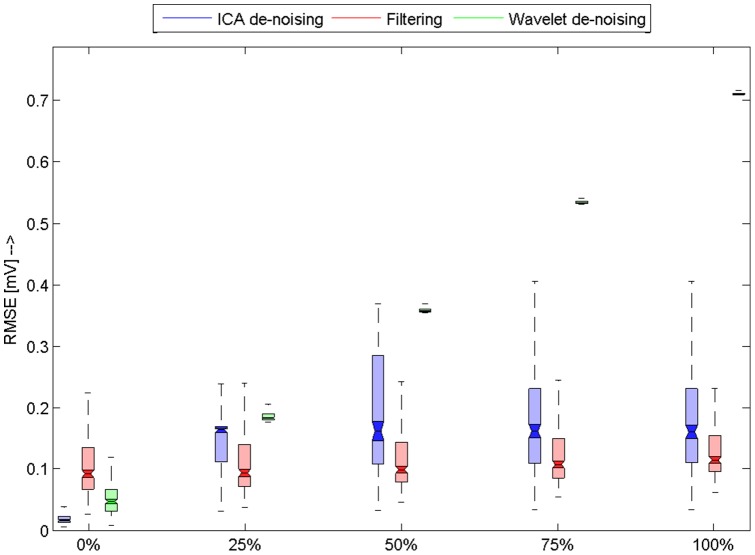
Results for the base line wander artefact. Horizontal axis shows level of added noise. Boxplots shows RMSE for used database of signals. Filtered methods that were used are differentiated by colors. We can observe that our algorithm achieves good results. The wavelet filtering algorithm has difficulties with this type of the artefact due to its simulation as slow sinus wave.

**Figure 9:**
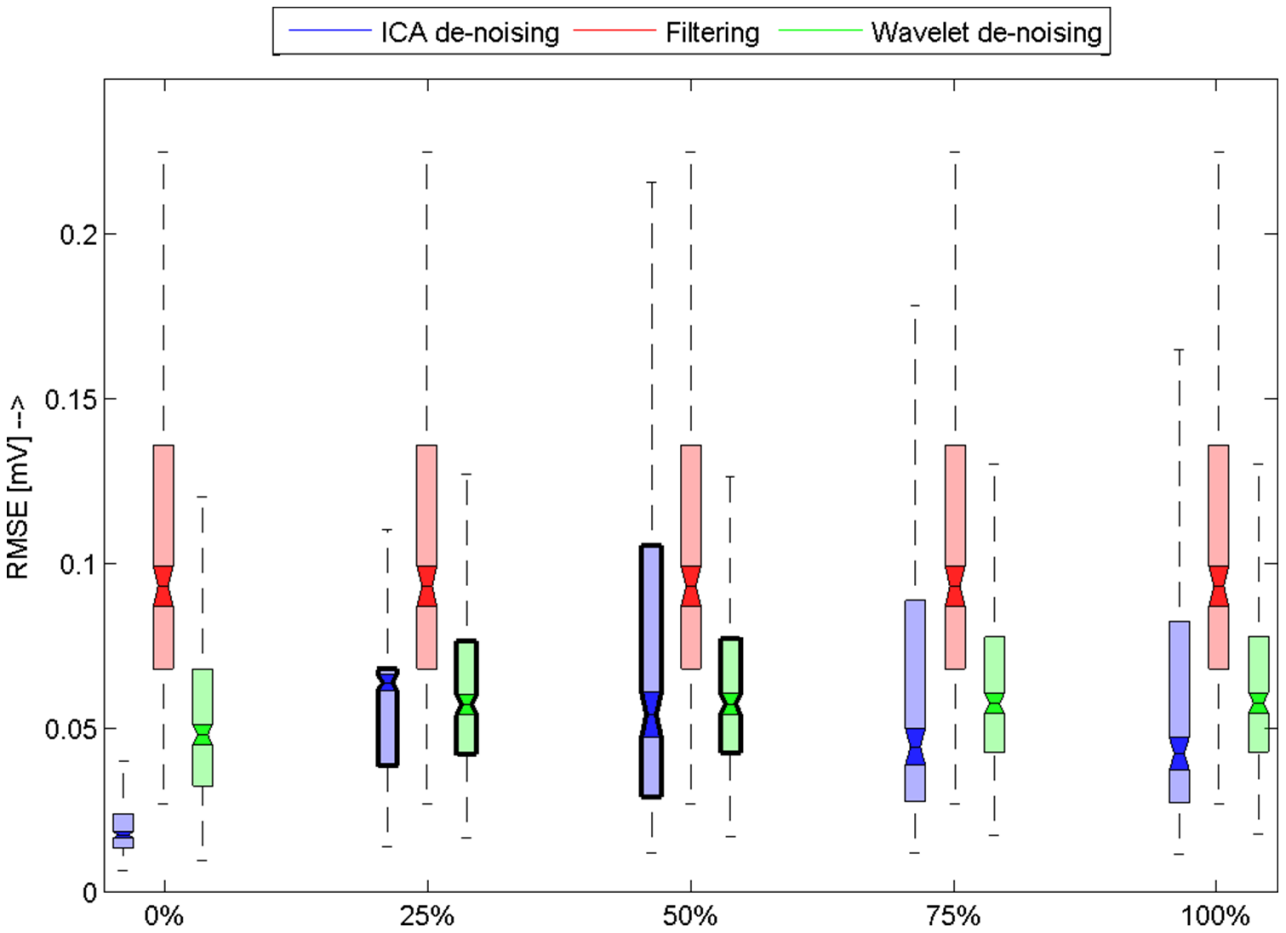
Results for the power line interference artefact. Horizontal axis shows level of added noise. Boxplots shows RMSE for used database of signals. Filtered methods that were used are differentiated by colors. Those boxplots where notches are marked with bold line around borders means that they means are not significantly different. We can observe that our algorithm works similarly to wavelet based de-noising and significantly better than filtering algorithm.

We performed statistical testing of 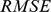 obtained as a result of comparison between original and filtered signals. The results are summarized using boxplot figures (Fig. 6, 7, 8, 9). Each figure contains 15 boxplots, which are linked to one type of noise. Boxplots are grouped according to the level of noise added to the original recording during the testing. Each boxplot has “notches”, which shows 95% confidence intervals for null hypothesis that 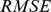 differs from others. If the confidence intervals are overlapping then we reject the null hypothesis. In addition to this visual evaluation we performed multicomparison Kruskal-Wallis test with Bonferroni correction in order to say that the results are different in general on 5% confidence level. To prove that the results differ on each level and for each type of noise we again performed Kruskal-Wallis test for each group of three 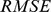
s, which corresponds to one noise and level. The obtained results are then evaluated using multicomparison technique using again Bonferoni correction. Null hypothesis was: Mean values differ on 95% confidence interval. Those results whose null hypothesis was rejected are marked with bold line around borders. That means if there is a group whose two boxplots are marked with bold borders they are not significantly different – the results of those algorithms are similar for selected noise type and level.

Figure 6 shows the results comparison on ECG contaminated by electrode cable artefact. We primarily developed this algorithm for removal of this type of artefact and we can observe that our algorithm outperforms both wavelet filtering and standard filtering across all noise levels added to the ECG signals (Fig. 6).

Figure 7 shows the results for ECG mixed with EMG. Again we can see that for all noise levels our algorithm outperform standard filtering and it works better or similar as wavelet filtering. We can observe that only for 100% noise level our algorithm works similar to wavelet filtering. We can observe that 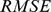 interquartile range of wavelet filtering and our algorithm are similar.

Figure 8 shows the results of ECG modulated by base line wander. We can observe that our algorithm is comparable but slightly worse than standard filtering. Both algorithms outperforms wavelet filtering. Wavelet filtering is unable to reduce base line wander artefact.

Finally figure 9 shows the results on recordings contaminated by power line interference. We can observe that our algorithm provide similar results as the comparison methods. ICA based algorithm has slightly better results than standard filtering and similar or better results than wavelet filtering. We can see that all 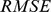 values are in 0.1 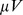 per sample, which is very good result anyway. Related to presented results we showed that our algorithm, which was originally designed to reduce electrode cable movement artefact, is capable to reduce power line interference comparably to standardized techniques.

## Discussion

Our data showed that suggested algorithm has significantly larger interquartile range than the other algorithms – this is because of two reasons. Firstly, suggested algorithm sometimes fails in identification of noise components and thus it does not reduce all noisy activity presented in ECG. This problem can be solved by extending the training set or changing the classification algorithm in order to enhance noise detection rate. Secondly and more significantly – the other methods do not reduce the noise at all, thus the 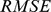 is quite constant across all recordings.

Wavelet filtering method is less effective with baseline wander artefact than the classical filtering (Fig. 8). This can be explained by the fact that the artefact was modelled as slow sine wave and the wavelet transform uses different basis for estimation of details and approximations, thus it is unable to efficiently reduce the base line wander. Standard filtering performed as the most effective because the filters in the set matches the frequency, which is used for simulation of baseline wander.

Problems of standard filtering with EMG type of artefact (Fig. 7) is due to spread of EMG across wide frequency range, thus the filtering techniques covering only narrow frequency range cannot reduce it efficiently.Our algoritm is more stable on the lower noise levelshowever in high noise levels keeps similar range in comparision to wavelet filtering.

Interestingly, in terms of noise mimicking ECG activity the JADE was very effective, because energies (in power spectra) and correlations of noise and ECG is high, thus the kurtosis (which is fourth order moment used by JADE for estimation of independent sources) enable to correct source separation [3]. Our approach combines BSS algorithm with detection step, which is similar to case in [16], [18]. Instead of searching for two types of noise (structured and unstructured) our method searches for the ECG containing components then it marks them and removes all other components. This approach seems to be useful, because ECG is regular and has very stable structure in sense of statistical parameters.

Suggested algorithm is the best of all in terms of “doing nothing”. These are cases where there is no noise in ECG signal. Such property of the algorithm is very crucial especially in biomedical signal applications, where in clinical practice one needs minimally distorted ECG as possible.

It is important to note that the decision tree trained only on portion of data proved to be perform similar when the method is applied on different ECG signals. This implies that a reasonable large database is needed (in our case MIT/BIH Arrhythmia Database containing 48 half-hour ECG recordings) for the training and then the pruned decision tree can be applied without any further change to other non-training data. Generally, however, if new type of noise needs to be filtered, the process of tree training needs to be repeated.

## Conclusions

We developed the noise reduction method based on Independent Component Analysis and decision tree, which first identifies noisy components and then it remove them from the signal. Suggested method is based on the method proposed in [3], which uses kurtosis of components to identify and remove noisy components from the signal containing EMG noise. The method introduces more sophisticated approach for identification of such noisy components and it generalizes the method so it is able to be be used to reduce various types of noises contaminating ECG signals. We also provided an extensive testing of designed method to prove its effectiveness. Suggested method has been proven on dataset containing 382 ECG recordings from various freely available databases. We also showed that the method, which was primarily designed to reduce electrode cable movement artefact, is capable to deal with different types of noises efficiently in comparison to regularly used ECG filtering approaches. The identification step of the algorithm gives the user the opportunity to adapt the method for his/her own purposes or to use different classifiers within it. Also the source separation algorithm can be changed in order to enhance abilities of the algorithm to deal with noise removal for different types of noise. This adaptability of the method will be part of next steps in our further research.

## Supporting Information

Appendix S1 **JADE algorithm.**
(PDF)

Appendix S2 **CART algorithm and pruning.**
(PDF)
