# Supplementary material for: Independent Component Analysis and Decision Trees for ECG Holter Recording De-Noising
Source: PLoS One. 2014 Jun 6;9(6):e98450. doi: 10.1371/journal.pone.0098450 (PMC4048160; doi:10.1371/journal.pone.0098450)
Supplement: Appendix S1 — JADE algorithm. (PDF) [file pone.0098450.s001.pdf]

Appendix 1 (S1): JADE algorithm

Joint Approximate Diagonalization of Eigen matrices algorithm($JADE$) is blind source separation method based on diagonalization of fourth-order cumulant tensor (we call it cumulant tensor for simplicity). Cumulant tensor is a four-dimensional array whose entries are given by fourth-order cross cumulants of data:

$$cum(x_i, x_j, x_k, x_l) = E\{x_i x_j x_k x_l\} - E\{x_i x_j\}E\{x_k x_l\} - E\{x_i x_k\}E\{x_j x_l\} - E\{x_i x_l\}E\{x_j x_k\}, \qquad (1)$$

where x_i represents measured mixture of source signals, $i, j, k, l = 1...n$, where n is number of measured mixtures and $E\{\}$ is expectation of data. Desirable property of cumulants is that all cumulants of linear combinations can be obtained as linear combinations of data cumulants [1]. Thus cumulants contain all fourth-order information of the data.

The cumulant tensor is a linear operator defined by the fourth-order cumulants defining linear transformation in the space of $n \times n$ matrices. We can define linear transformation on linear space of dimension $n \times n$. The i, jth element of matrix given by transformation $\mathbf{F}_{ij}$ is defined as:

$$\mathbf{F}_{ij}(\mathbf{M}) = \sum_{kl} m_{kl} cum(x_i, x_j, x_k, x_l), \qquad (2)$$

where m_{kl} are the elements of transformed matrix $\mathbf{M}$.

Cumulant tensor as any symetric operator has an eigenvalue decomposition. An eigenmatrix of the tensor is a matrix such that:

$$\mathbf{F}(\mathbf{M}) = \lambda \mathbf{M}, \qquad (3)$$

where λ is a scalar eigenvalue.

Considering whitened data: $\mathbf{z} = \mathbf{Vx} = \mathbf{VAs} = \mathbf{W}^T\mathbf{s}$, where $\mathbf{z}$ is whitened data, $\mathbf{V}$ is whitening matrix, $\mathbf{x}$ are mixed signals, $\mathbf{A}$ is mixing matrix and $\mathbf{s}$ are desired source signals, the cumulant tensor of $\mathbf{z}$ has special structure that can be seen in the eigenvalue decomposition. Every matrix of the form:

$$\mathbf{M} = \mathbf{w}_m \mathbf{w}_m^T, \qquad (4)$$

for $m = 1, ..., n$ is an eigenmatrix. The vector $\mathbf{w}_m$ is one of the rows of matrix $\mathbf{W}$. It can be shown that:

$$\mathbf{F}_{ij}(\mathbf{w}_m \mathbf{w}_m^T) = w_{mi} w_{mj} kurt(s_m), \qquad (5)$$

where $kurt(s_m)$ is kurtosis of corresponding source signal s_m. This is because all elements of cumulant tensor, whose indices are different equal zero due to independence of source signals [1]. We can observe that if we knew eigenmatrices we could easily obtain independent components.

All above can be rephrased as follows: The matrix $\mathbf{W}$ diagonalizes $\mathbf{F}(\mathbf{M})$ for any $\mathbf{M}$. This means that $\mathbf{WF}(\mathbf{M})\mathbf{W}^T$ is diagonal. This is because matrix $\mathbf{F}$ is diagonal for linear combination of terms of form $\mathbf{w}_i \mathbf{w}_i^T$ assuming that ICA model holds. Thus we can take set of different matrices $M_i, i = 1, ..., k$ and try to make $\mathbf{WF}(\mathbf{M})_i \mathbf{W}^T$ as diagonal as possible. In real situation the model does not hold exactly and errors in data make the exact diagonalization impossible.

During diagonalization one needs to measure diagonality of matrix $\mathbf{WF}(\mathbf{M})_i \mathbf{W}^T$, this can be measured i.e. using the following measure [1]:

$$J_{JADE}(\mathbf{W}) = \sum_i ||diag(\mathbf{WF}(\mathbf{M})_i \mathbf{W}^T)||^2, \qquad (6)$$

where $||diag()||^2$ means sum of squares of the diagonal.

The crucial is selection of set of matrices $\mathbf{M}_i$. One possible and natural choice is to take the eigenmatrices of the cumulant tensor, which is exactly what JADE algorithm does [1].

References

1. Hyvärinen A, Karhunen J, Oja E (2001) Independent Component Analysis. Wiley.
