# Supplementary material for: Independent Component Analysis and Decision Trees for ECG Holter Recording De-Noising
Source: PLoS One. 2014 Jun 6;9(6):e98450. doi: 10.1371/journal.pone.0098450 (PMC4048160; doi:10.1371/journal.pone.0098450)
Supplement: Appendix S2 — CART algorithm and pruning. (PDF) [file pone.0098450.s002.pdf]

Appendix 2 (S2): CART algorithm and pruning

Classification And Regression Trees (CART) algorithm [1] is a classification algorithm for building a decision tree based on Gini's impurity index as splitting criterion. CART is a binary tree build by splitting node into two child nodes repeatedly. The algorithm works repeatedly in three steps:

1. Find each feature's best split. For each feature with K different values there exist K-1 possible splits. Find the split, which maximizes the splitting criterion. The resulting set of splits contains best splits (one for each feature).
2. Find the node's best split. Among the best splits from Step i find the one, which maximizes the splitting criterion.
3. Split the node using best node split from Step ii and repeat from Step i until stopping criterion is satisfied.

As splitting criterion we used Gini's impurity index, which is defined for node t as:

$$i(t) = \sum_{i,j} C(i|j)p(i|t)p(j|t), \tag{1}$$

where $C(i|j)$ is cost of misclassifying a class j case as a class i case (in our case $C(i|j) = 1$, if $i \neq j$ and $C(i|j) = 0$ if $i = j$), $p(i|t)$ ($p(j|t)$ respectively) is probability of case in class i (j) given that falls into node t.
The Gini impurity criterion is type of decrease of impurity, which is defined as:

$$\Delta i(s,t) = i(t) - p_L i(t_L) - p_R i(t_R), \tag{2}$$

where $\Delta i(s,t)$ is decrease of impurity at node t with split s, p_L (p_R) are probabilities of sending case to the left (right) child node t_L (t_R) and $i(t_L)$ ($i(t_R)$) is Gini impurity measure for left (right) child node.
In order to enhance generalization of decision tree we used pruning with combination of cross-validation error rate estimation. The algorithm for pruning works as follows [1]:

1. Split randomly training data into 10 folds.
2. Select pruning level of tree (level 0 equals to full decision tree).
3. Use 9 folds for creation of 9 new pruned trees and estimate error on last 10th fold.
4. Repeat from Step ii until all pruning levels are used.
5. Find the smallest error and use the pruning level assigned to it.
6. Until pruning level is reached, remove all terminal nodes in the lowest tree level and assign decision class to parent node. Decision value is equal to class with higher number of cases covered by node.

References

1. Breiman L (1984) Classification and regression trees. The Wadsworth and Brooks-Cole statistics-probability series. Chapman & Hall.
